# Evaluation of fibrosis in cirrhotic elderly patients with HCC

**DOI:** 10.1186/1471-2318-11-S1-A47

**Published:** 2011-08-24

**Authors:** A Pesce, MA Trovato, A Branca, R Scilletta, TR Portale, S Puleo

**Affiliations:** 1 Department of General Surgery, University of Catania, AOU Policlinico-Vittorio Emanuele, Catania, Italy

## Background

Hepatocellular carcinoma (HCC) is usually associated with liver cirrhosis and is the principal cause of death among patients with cirrhosis [1]. Apart from liver transplantation that may cure both conditions, treatment of HCC and cirrhosis is complex because of the need to be oncologically radical but simultaneously conservative. Hepatectomy is considered an invasive approach and has a marginal role in the treatment of HCC [2,3]. The value of hepatic fibrosis is considered a predictive factor of outcome in patients with HCC undergoing liver resection [4].

## Materials and methods

A retrospective analysis of 77 cirrhotic patients, 42 of them with hepatocellular carcinoma, observed from 2008 to 2010 was performed. The mean age was 65.2 years old with 46 men and 31 women. As regards cirrhosis etiology, 51 patients presented cirrhosis HCV-related, 9 HBV-related, 3 alcohol-related, 2 HBV-HDV co-infections and 12 other etiology. Liver function was assessed according to the Child-Pugh classification: 60 patients were in Child A, 13 in Child B and 4 in Child C. In all patients liver stiffness measurement (LSM) was performed using transient elastography (Fibroscan^®^). Forty patients were over 65 years old, thirty-seven under 65 ys.

## Results

The mean value of liver stiffness was 27.9 kPa (F4). In the group of elderly patients the mean value of liver stiffness was 25.2 kPa versus 31.2 kPa of the group of patients under 65 ys. In the elderly group 37 patients were in Child A, 5 in Child B and one in Child C. In the other group 22 patients were in Child A, 8 in Child B and 3 in Child C.

## Conclusions

These results confirm that the severity of liver fibrosis is directly associated with liver function (Child class) and not with the age of patients. Pre-operative liver stiffness measurement in cirrhotic elderly patients with HCC and Child class A could help us in the evaluation of surgical risk in order to reduce post-operative morbidity and mortality.
